# Medical News

**Published:** 1851-03

**Authors:** 


### MEDICAL NEWS.

CASE OF FISTULA OF THE STOMACH.—Another case of communication between the stomach and external surface of the abdomen, very similar to that of Alexis St. Martin, the well known subject of Dr. Beaumont's interesting observations, is reported by Dr. Robertson, of the Edinburgh Royal Infirmary, in the Edinburgh Monthly Journal of Medical Science, for January, 1851. The perforation had resulted from internal causes, and the present condition of the patient, a woman, thirty-six years of age, encourages the hope of some valuable results from experiments on the digestive process. Drs. Christison, Syme, and others, have been appointed, on behalf of the Edinburgh Medico-Chirurgical Society, to assist Dr. Robertson in his investigations.

We learn that Prof. HORNER, of the University of Pa., has, in a recent dissection, distinctly seen the *Pleuro-Œsophageal* and *Broncho-Œsophageal Muscles,* as described by Prof. Hyrtl, of Vienna.

DEATHS OF DISTINGUISHED PHYSICIANS.—France has recently lost one of her most eminent physicians in the person of M. FOUQUIER, physician to La Charité.

Dr. John HAVILAND, Regius Professor of Medicine in the University of Cambridge, England, died on the eighth of January last.

DELEGATES TO THE AMERICAN MEDICAL ASSOCIATION.—At a Meeting of the College of Physicians, of Philadelphia, held 4th February, 1851, the following gentlemen were elected delegates to the Meeting of the Association, to be held at Charleston, in May next. Drs. G. B. Wood, G. Emerson, G. W. Norris, W. S. W. Ruschenberger, J. R. Paul, F. G. Smith, D. F. Condie, I. Hays, W. B. Page, W. Pepper, J. Carson, J. Neill, and Caspar Morris.

The Delegates from the County Medical Society have not yet been chosen.

DR. JOHN CURWEN has been elected Superintendant of the State Lunatic Asylum of Pennsylvania.

The Catalogue of the University of Pa. announces 466 Students for the current session. That of the Jefferson Medical College, 504.

The population of Virginia, according to the recent census, numbers 1,428,000, of which 1755 are Physicians—giving a ratio of one Physician to every 813 inhabitants.

PROFESSOR OF CHEMISTRY IN HARVARD UNIVERSITY.—We learn that Mr. S. P. Cook, the Professor of Mineralogy, &c., in Harvard University, has been appointed Professor of Chemistry in place of Professor Horsford, and will enter upon his duties next fall. In the mean time he will make a tour through Europe for scientific improvement.

ADULTERATED DRUGS IN NEW YORK.—It is stated in the Newark Daily Advertiser, that Dr. M. J. Bailey, the first Examiner of Drugs for the port of New York, during nine months prior to his removal from office, rejected 90,000 pounds of various base drugs. For some reason, unknown to us, Dr. B. has been displaced. It is stated in the paper above alluded to, also in one or more of the New York papers, that efforts have been made for the restoration of Dr. B., on the ground of his superior qualifications, and the increased importation of adulterated drugs since his displacement.—*Bost. Med. and Surg. Journ.*

ASSISTANT SURGEONS UNITED STATES NAVY.—The following Assistant Surgeons in the Navy, examined by the Medical Board convened at the Naval Asylum, Philadelphia, have been found qualified for promotion, and passed, viz :—

Robert T. Macoun, Passed Assistant Surgeon, to rank next after Passed Asistant Surgeon Richard McSherry.

William A. Harris, Passed Assistant Surgeon, to rank next after Passed Assistant Surgeon Robert E. Wall.

Passed Assistant Surgeon Henry O. Mayo, to rank next after Passed Assistant Surgeon Wm. A. Harris.

Of the candidates examined for admission into the service as Assistant Surgeons, the following have been found qualified, viz :—

No. 1. Samuel F. Cowes, Portsmouth, N. H.
No. 2. Jacob S. Dungan, Philadelphia.
No. 3. George Peck, New York.
No. 4. Charles F. Fahs, York, Pennsylvania.
No. 5. Jenks H. Otis, Boston.
No. 6. Frederick Horner, Jr., Warrenton, Va.
No. 7. James B. Whiting, Norfolk, Va.
No. 8. Randolph Harrison, Cartersville, Va.
No. 9. W. E. Wysham, Baltimore, Md.
No. 10. Albert Schriver, Philadelphia, Pa.
No. 11. Thomas Le Page Cronmiller, Savage Factory, Md.
No. 12. E. Drayton, Philadelphia, Pa.
No. 13. Wm. L. Nichol, Nashville, Tenn.
No. 14. John C. Coleman, Halifax Courthouse, Va.
No. 15. J. Page Hopkins, Winchester, Va.
No. 16. Richard H. Cowman, Annapolis, M.

